# Basal core promoter T1762/A1764 and precore A1896 gene mutations in hepatitis B surface antigen-positive hepatocellular carcinoma: a comparison with chronic carriers

**DOI:** 10.1111/j.1478-3231.2007.01585.x

**Published:** 2007-12

**Authors:** Myron J Tong, Lawrence M Blatt, Jia-Horng Kao, Jason Tzuying Cheng, William G Corey

**Affiliations:** 1 The Liver Center, Huntington Medical Research Institutes Pasadena, CA, USA; 2 The Pfleger Liver Institute, David Geffen School of Medicine at the University of California in Los Angeles Los Angeles, CA, USA; 3 Intermune Inc. Brisbane, CA, USA; 4 The Hepatitis Research Center, National Taiwan University Hospital Taipei, Taiwan

**Keywords:** basal core promoter mutants, chronic carriers, HBV DNA, HBV genotypes, hepatocellular carcinoma, precore mutants

## Abstract

**Background:**

Chronic hepatitis B virus (HBV) infection is associated with hepatocellular carcinoma (HCC), and specific viral factors have been identified that may increase the risk for HCC development. However, the differences in these viral factors in chronic carriers who seldom develop HCC compared with HCC patients have not been adequately evaluated.

**Methods:**

From 1989 to 2005, 101 hepatitis B surface antigen-positive patients presented to our clinic with HCC. Baseline basal core promoter (BCP) T1762/A1764 mutants, precore (PC) A1896 mutants, HBV genotypes and HBV DNA in HCC patients were compared with 67 chronic carriers who had been followed for a mean of 112.1±77.7 standard deviation months.

**Results:**

At baseline, HCC patients had lower levels of serum albumin, but higher values of alkaline phosphatase, aspartate aminotransferase, alanine aminotransferase, bilirubin and α-foetoprotein than those of chronic carriers (*P* < 0.001 for all comparisons). The presence of genotype C, higher frequencies of PC A1896 mutants, BCP T1762/A1764 mutants and higher circulating levels of HBV DNA were more frequently detected in HCC patients than that in chronic carriers (*P* < 0.001 for all observations). Logistic regression analysis revealed that BCP T1762/A1764 mutants [odds ratio (OR) 11.14, 95% confidence interval (CI) 3.05–40.72; *P* < 0.001] and PC A1896 mutants (OR 3.75, 95% CI 1.14–12.34; *P* < 0.05) were significantly associated with HCC development.

**Conclusion:**

Our results indicate that the presence of BCP and PC mutations significantly increases the risk for HCC in chronic hepatitis B patients. These mutations were less often detected in chronic carriers who seldom develop HCC.

Hepatocellular carcinoma (HCC) is a common malignancy in many areas of the world (1). The largest concentrations of HCC cases are in Asia and in Africa, where over 80% of liver cancer cases are reported. In these countries, the most important aetiology for HCC is chronic hepatitis B virus (HBV) infection, and strong geographical correlations have been reported between the incidence of HCC and the prevalence of HBV infections in these areas.

The development of chronic HBV infection is dependent on the age of HBV exposure. Up to 90% of infants born to hepatitis B surface antigen (HBsAg)-positive and hepatitis B e antigen (HBeAg)-positive mothers, and 5–10% of adults with acute HBV infection progress to chronic hepatitis B. Thereafter, it is estimated that 15–40% of chronic hepatitis B patients will progress to cirrhosis and to HCC (2). This indicates that 60–85% of HBsAg-positive carriers will have a benign outcome and may remain as inactive carriers with no progression of liver disease. Thus, it is important to identify HBV-infected patients who are at a higher risk for disease progression from those who will not develop decompensated liver disease or HCC.

The reasons why some patients with chronic hepatitis B infection progress to HCC are unknown. Host factors such as an immune response to HBV, a genetic predisposition to HCC development, high HBV replication rates, as well as mutations within the HBV genome have been related to HCC development. A dual mutation in the basal core promoter (BCP) region of the HBV genome involving an A–T substitution at nucleotide 1762 and a G–A substitution at nucleotide 1764 has been associated with HCC development (3–5). Also, a mutation in the precore (PC) region of the HBV genome involving a G–A substitution at nucleotide 1896 has been described in patients with HBeAg-negative chronic hepatitis (6). However, its role in HCC pathogenesis is not well established. Studies from Asia have indicated that patients with genotype C are at a higher risk for HCC than patients with other genotypes (7–10). Also, genotype B has been implicated in a younger population of HCC in Taiwan, but in older HCC patients in Japan (7, 8, 11, 12). Recently, elevated serum levels of HBV DNA have been noted to increase the risk for HCC (13–15).

In the present report, we compared the baseline hepatitis B virological profiles of 67 chronic carriers who had been followed for a mean of 9 years with no liver complications with 101 HCC patients who were referred to our Liver Center. Our aim was to identify differences in the BCP and PC mutations, HBV genotypes and baseline HBV DNA levels between these two groups of patients who had significantly different clinical outcomes.

## Methods

### Patients

From January 1989 to June 2005, 101 patients with HBsAg-positive HCC were referred to our Liver Center in Pasadena, CA, USA. The diagnosis of HCC was confirmed by biopsy of the mass lesion in the liver, and by typical computed tomography or magnetic resonance imaging findings for HCC along with α-foetoprotein (AFP) serum levels of ≥20 ng/mL. Also, from January 1989 to December 1998, 110 HBsAg-positive patients who were classified as chronic carriers were enrolled in a prospective study (16). At presentation, these chronic carriers had no symptoms or signs of chronic liver disease, and had normal liver tests and normal platelet counts. These chronic carriers visited our clinic every 6–12 months and had persistently normal liver tests. During a mean follow-up of 112.1±77.7 standard deviation (SD) months, none had progression of liver disease or HCC development. In order to conform to the standard definition of a chronic carrier (17–19), we selected from this group of patients only those who were HBeAg negative, anti-HBe positive, had persistently normal serum aspartate aminotransferase (AST) and alanine aminotransferase (ALT) levels and had baseline serum HBV DNA levels of ≤10^5^ copies/mL that were measured in one laboratory in 2005 as described below. Sixty-seven of the original 110 patients fit the standard criteria for a chronic carrier, and they were used for comparison with the HCC patients. In these chronic carriers, 63 had baseline HBV DNA values of <100 000 copies/mL while four patients had HBV DNA levels of 141 000, 150 000, 160 000 and 335 000 copies/mL respectively. For both chronic carriers and HCC patients, a serum sample was drawn during the initial visit and stored at −70° until use.

### Laboratory tests

During the initial visit, measurements of serum albumin, AST, ALT, total bilirubin, alkaline phosphatase, platelet counts and AFP levels were obtained from 101 HCC patients and from 67 chronic carriers. HBeAg and anti-HBe were measured with commercially available kits (Abbott Laboratories, North Chicago, IL, USA). In 2005, we collaborated with the Hepatitis Research Center at National Taiwan University Hospital in Taipei, Taiwan, and stored serum samples from the 101 HCC patients, and 67 chronic carriers were sent for analysis of BCP mutants, PC mutants, HBV genotypes and HBV DNA.

Serum HBV DNA was quantified by a real-time polymerase chain reaction assay in the linear range from 10^2^ to 10^11^ copies/mL (20). For reporting purposes, the HBV DNA values were log_10_ transformed. The identification of HBV genotypes was performed by melting curve analysis (20), and supplemented by direct sequencing of the pre-S amplicon and phylogenetic analysis by comparing the nucleotide sequence with 33 reference HBV strains obtained from GenBank as described previously (21). Amplification and sequencing of PC (nucleotides 1814–1900) and BCP (nucleotides 1742–1849) genes were performed as described (5, 7). The nucleotide sequences of the amplified products were directly determined using fluorescence-labelled primers with a 3100 Automatic Sequencer (Applied Biosystems, Foster City, CA, USA). Sequencing conditions were specified in the protocol for the Taq DyeDeoxy Terminator Cycle Sequencing Kit (Applied Biosystems). The inner primer pair was used as sequencing primers for both directions of the gene.

### Statistical analysis

We investigated the distribution of different baseline characteristics by HCC status. All continuous variables with a normal distribution were compared using Student's *t*-test. In case of a non-normal distribution, the data were normalized with log transformation before *t*-tests. Categorical data were compared using χ^2^ tests.

Initially, we performed χ^2^ tests to investigate the relationship between the possible risk factors of HCC, i.e. genotype C infection, BCP T1762/A1764 and PC A1896 gene mutations. Both BCP and PC mutations were coded as dominant. We then fitted a logistic regression model for each of those potential risk factors, adjusting for age, sex and race (partially adjusted model). The possible confounding effect between the risk factors was evaluated by fitting logistic models with any combination of the two risk factors and noting more than a 10% change in the odds ratios (OR) compared with the single risk factor models. Effect modifications between the risk factors were evaluated by fitting models with proper interaction terms and performing likelihood ratio tests between the partial (model with the main effect of variables of interest) and full model (model with the main effect of both variables as well as the proper interaction term). The fully adjusted model for HCC selected was based on the factors identified in the process described above and by comparing different models using Akaike information criterion (AIC) (22). The lowest AIC identified the best model. All analysis was performed using sas 9.1.

## Results

### Patients and laboratory tests

At presentation, the mean age of the HCC patients was older than the chronic carriers (53.3±13.5 vs. 45.4±12.3 years, *P* = 0.002), and more HCC patients were males (83.2 vs. 43.3%, *P* < 0.0001; Table 1). The HCC patients had significantly lower baseline mean serum albumin values but higher AST, ALT, alkaline phosphatase and total bilirubin levels than chronic carriers (*P* < 0.0001 for all observations). Also, the mean serum AFP values were significantly higher in HCC patients than in chronic carriers (*P* = 0.0001).

**Table 1:** Baseline characteristics of hepatitis B surface antigen-positive chronic carriers and hepatocellular carcinoma patients

	Chronic carriers	HCC patients	*P* value
Number of patients	67	101	
Age (mean)	45.4 ± 12.3 years	53.3 ± 13.5 years	0.002
Male	29 (43.3%)	84 (83.2%)	<0.0001
Asian	58 (86.6%)	91 (90.1%)	0.35
Alkaline phosphatase (mean)	66.5 ± 50.2 U/L	201.3 ± 288.3 U/L	<0.0001
Albumin (mean)	4.4 ± 0.4 mg/dL	3.7 ± 0.7 mg/dL	<0.0001
AST (mean)	18.7 ± 11.4 U/L	128.8 ± 142.6 U/L	<0.0001
ALT (mean)	19.9 ± 13.8 U/L	92.9 ± 99.4 U/L	<0.0001
Total bilirubin (mean)	0.52 ± 0.23 mg/dL	1.7 ± 2.6 mg/dL	<0.0001
Platelets (mean)	221.6 ± 94.5 × 10^3^/mm^3^	203.8 ± 149.2 × 10^3^/mm^3^	0.37
AFP (median)	0.39 (minimum 0, maximum 0.9) log_10_ ng/mL	2.15 (minimum 0, maximum 6.25) log_10_ ng/mL	0.0001
AFP (mean)	0.39 ± 0.18 log_10_ ng/mL	2.44 ± 1.55 log_10_ ng/mL	0.0001

AFP, α-foetoprotein; ALT, alanine aminotransferase; AST, aspartate aminotransferase; HCC, hepatocellular carcinoma.

### Hepatitis B virological tests

Patients with HCC had a higher prevalence of genotype C than chronic carriers (68.1 vs. 34.2%, *P* = 0.0003), while genotype B was more frequently detected in chronic carriers than in HCC patients (48.8 vs. 24.5%, *P* = 0.006; Table 2). Compared with chronic carriers, HCC patients had a significantly higher frequency of BCP T1762/A1764 mutants (77.7 vs. 21.2%, *P* = 0.0001; Table 3), and a higher frequency of PC A1896 mutants (46.0 vs. 30.2%, *P* = 0.04).

**Table 3:** Demographic and virologic characteristics of hepatitis B virus chronic carriers and patients with hepatocellular carcinoma

	Chronic carriers	Patients with HCC		
Characteristics*	*n* = 67	*n* = 101	*P* value	OR (95% CI)†
Age	45.4 ± 12.3 SD years	53.3 ± 13.5 SD years	0.002	1.05 (1.02–1.08)
Gender				
Female	38 (56.7%)	17 (17.0%)	0.0001	1
Male	29 (43.3%)	84 (83.0%)		6.47 (3.18–13.18)
Asian				
No	9 (13.4%)	9 (8.9%)	0.35	1
Yes	58 (86.6%)	92 (91.1%)		1.59 (0.59–4.23)
HBV genotype C				
No	27 (65.8%)	30 (31.9%)	0.0003	1
Yes	14 (34.2%)	64 (68.1%)		4.11 (1.89–8.95)
PC				
Wild type	44 (69.8%)	54 (54.0%)	0.04	1
A1896 mutant	19 (30.2%)	46 (46.0%)		1.97 (1.01–3.84)
BCP				
Wild type	37 (78.7%)	21 (22.3%)	0.0001	1
T1762/A1764 mutant	10 (21.2%)	73 (77.7%)		12.86 (5.49–30.11)
HBV load				
≤10^5^ copies/mL	67 (100.0%)	63 (51.5%)	0.0001	NA
>10^5^ copies/mL	0 (0.0%)	38 (48.5%)		

* Data are provided as *n* (%) or mean ± SD.

† When *n* (%) is provided, the *P* value and OR (95% CI) is reported from χ^2^ tests or Fisher's exact test. In case of mean ± SD, the *P* value is reported from *t*-tests and OR (95% CI) is reported from univariate logistic regression.

BCP, basal core promoter; CI, confidence interval; HBV, hepatitis B virus; HCC, hepatocellular carcinoma; NA, not applicable; OR, odds ratio; PC, precore; SD, standard deviation.

**Table 2:** Baseline virologic markers in hepatitis B surface antigen-positive chronic carriers and hepatocellular carcinoma patients

	Chronic carriers	HCC patients	*P* value
HBV genotype			
A	4/ 41 (9.8%)	4/ 93 (4.3%)	NS
B	20/41 (48.8%)	23/93 (24.7%)	0.02
C	13/41 (31.7%)	62/93 (66.7%)	0.02
A+B	0/ 41 (0.0%)	1/ 93 (1.1%)	NS
B+C	1/ 41 (2.4%)	2/ 93 (2.1%)	NS
D	3/ 41 (7.3%)	1/ 93 (1.1%)	NS
HBV DNA (mean)	2.56 ± 1.99 log_10_ copies/mL	5.97 ± 1.63 log_10_ copies/mL	<0.0001

HBV, hepatitis B virus; HCC, hepatocellular carcinoma; NS, not significant.

**Fig. 1:**
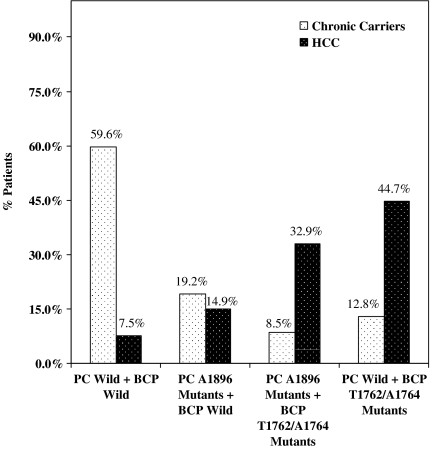
Analysis of precore and basal core promoter wild types and mutants in chronic carriers and hepatocellular carcinoma (HCC) patients.

We next analysed for the presence of combinations of either PC wild type or A1896 mutant sequences and BCP wild type or T1762/A1764 mutant sequences in the HCC patients and chronic carriers. As can be seen in Figure 1, there were significantly more PC wild-type sequences plus BCP wild-type sequences in the chronic carriers than that in the HCC patients. Also, compared with chronic carriers, higher numbers of PC wild-type sequences plus BCP T1762/A1764 mutant sequences (44.7 vs. 12.8%), and more PC A1896 mutant sequences plus BCP T1762/A1764 mutant sequences were detected in HCC patients (32.9 vs. 8.5%, *P* = 0.001 for all observations).

The mean baseline serum HBV DNA values in the HCC patients were significantly higher compared with chronic carriers who were preselected to have serum HBV DNA levels of ≤10^5^ copies/mL (*P* < 0.0001; Table 2). However, over 50% of the HCC patients also had HBV DNA levels of ≤10^5^ copies/mL (Table 3). In 98 HCC patients tested, 29 (29.6%) were HBeAg positive, 68 (69.4%) were anti-HBe positive and one (1%) was both HBeAg positive and anti-HBe positive. By preselection, all 67 chronic carriers were HBeAg negative and anti-HBe positive.

By univariate analysis, older age (*P* = 0.002), male gender (*P* = 0.001), HBV genotype C (*P* = 0.0003), PC A1896 mutants (*P* = 0.04) and BCP T1762/A1764 mutants (*P* = 0.0001) were significantly associated with HCC (Table 3).

### Logistic regression models

The HBV genotype was not detectable in 33 patients, and another nine patients did not have measurable BCP sequences. Thus, a total of 126 patients had complete data for HBV molecular markers. Logistic regression analysis was conducted utilizing unrestricted (*n* = 168) and restricted (*n* = 126) data sets to determine the sensitivity of HBV molecular markers as risk factors for HCC, and both models yielded similar results. Next, logistic regression analysis was conducted with partially adjusted models (age, gender and race) and with a model fully adjusted for all the risk factors (age, sex, race, HBV genotype, PC A1896 mutation, BCP T1762/A1764 mutation). The latter was the best predictor model because it had the lowest AIC (106.3). Thus, utilizing the fully adjusted model, we found that the BCP T1762/A1764 mutation was independently associated with HCC development [OR 11.14, 95% confidence interval (CI) 3.05–40.72; *P* < 0.001; Table 4]. In addition, the PC A1896 mutation was also associated with a higher risk for developing HCC (OR 3.75, 95% CI 1.14–12.34; *P* < 0.05). However, the HBV genotype was not an independent predictor for HCC.

**Table 4:** Multivariate analysis of viral factors associated with the development of hepatocellular carcinoma*

	OR (95% CI)	*P* value
HBV genotype C		
No	1	
Yes	3.28 (0.89–12.13)	<0.10
PC A1896 mutation		
No	1	
Yes	3.75 (1.14–12.34)	<0.05
BCP T1762/A1764 mutation		
No	1	
Yes	11.14 (3.05–40.72)	<0.001

* Analyses restricted to 126 subjects (HCC=90, chronic carriers=36) who had complete tests on HBV genotype (genotype C), PC and BCP genes. The multivariate model was adjusted for age, gender and race. BCP, basal core promoter; CI, confidence interval; HBV, hepatitis B virus; HCC, hepatocellular carcinoma; OR, odds ratio; PC, precore.

### Follow-up

During a mean follow-up period of 112.1±77.7 SD months, none of the 67 chronic carriers had progression of liver disease or developed HCC. In the 101 patients with HCC, nine received liver transplantation, 19 had liver resection, 24 had transarterial chemoembolization, six had percutaneous ethanol injection, three had radiofrequency ablation and 42 patients received no therapy. Only nine HCC patients are still alive at this time, but three have recurrence of HCC.

## Discussion

The average age of our HBsAg-positive HCC patients at presentation was 53 years and is similar to HCC patients from China, Taiwan and Japan (3, 7, 12). Also, 83% of our HCC patients were male, which also is observed worldwide (1). This is in contrast to our chronic carriers in which over 50% were female. Although the mean age of our chronic carriers was 41.2 years on presentation, their average age was 50 years at last follow-up, which is close to the mean age of our HCC patients at their first clinic visit. During the average 112 months of follow-up, none of the chronic carriers had progressive liver disease or developed HCC, suggesting that their prognosis is excellent (23). If this is the case, these chronic carriers may not require antiviral treatment. However, the incidence of HCC occurrence in chronic carriers is estimated to be 0.1%, and so annual surveillance for HCC is still recommended (24). In our previous report, all laboratory parameters of liver function in HCC patients were abnormal, but none of these tests was able to distinguish HCC patients from those with HBV-related cirrhosis (16).

Previous studies have indicated that high circulating HBV DNA levels are predictive for liver disease progression. In an 11-year follow-up study of 3582 Chinese patients, a high viral load at entry was associated with progression to cirrhosis (25). Also, we recently reported that cirrhosis patients with elevated HBV DNA levels had a higher probability of death from non-HCC-related liver complications than those with low HBV DNA values (26). High-circulating HBV DNA levels were also predictive for HCC development (3, 13, 15). In a report from Taiwan, the cumulative incidence rate for HCC was 1.3% in patients with baseline HBV DNA levels of <300 copies/mL compared with a 14.9% incidence rate in patients with ≥10^6^ copies/mL (15). In the latter study, patients with persistent HBV DNA elevations during follow-up had the highest HCC risk. In the present study, we preselected an HBV DNA level of ≤10^5^ copies/mL in the chronic carriers, and none of these patients had liver disease progression or developed HCC. However, 51% of our HCC patients presented with HBV DNA levels of ≤10^5^ copies/mL. Because we preselected the HBV DNA level in the definition of our chronic carriers, we were unable to make meaningful comparisons with the HCC patients.

The mutation from G to A in the PC region at nucleotide 1896 was described in patients with HBeAg-negative chronic hepatitis (6, 27). This PC A1896 mutation creates a stop codon that prevents translation of the PC protein and abolishes the production of HBeAg. However, these patients continue to synthesize HBV DNA at sufficient levels to cause continual liver damage with progression to cirrhosis (21). The frequency of PC mutants has been reported to be similar in patients with HCC and in inactive carriers, and some authors even suggested that acquisition of PC mutants may actually lead to inactivation of the chronic liver disease state (5, 28). In our study, the presence of PC mutants alone was detected in 30.2% of the chronic carriers and 46% of the HCC patients (*P* = 0.04). Also, 33% of our HCC patients had both PC A1896 and BCP A1762/T1764 mutants, while 15% of the HCC patients had PC A1896 mutants and BCP A1762/T1764 wild-type sequences (Fig. 1). This observation suggests that the BCP A1762/T1764 mutation may be the principal driving force for the development of HCC, and the PC A1896 mutants also play a significant, albeit lesser, role in the progression to HCC. Up until the present, PC A1896 mutants have not been implicated in HCC development. To further clarify the role of PC A1896 mutants in HCC development, future studies should include other viral factors such as genotypes and BCP A1762/T1764 mutants as possible confounding factors.

The BCP T1762/A1764 mutations were detected in HCC patients from Asia and Africa. A study from Guangxi Autonomous Region in China showed that close to 100% of HCC patients tested had BCP T1762/A1764 mutants in both serum and tumour tissue (3). Another report from Taiwan showed that 71% of HCC patients had BCP T1762/A1764 mutants (5). The prevalence of BCP T1762/A1764 in African patients with HCC was 66% compared with only 11% in asymptomatic carriers (4). Recently, we reported that the presence of genetic mutations in the BCP and PC regions in cirrhosis patients was predictive for HCC development (26). Other studies identified the role of BCP mutants with high HBV DNA levels in hepatocarcinogenesis (29, 30). In the present study, BCP T1762/A1764 mutants alone or in combination with PC A1896 mutants were detected in 45 and 33% of our HCC patients respectively. Our findings emphasize the important role of the BCP T1762/A1764 mutants in the pathogenesis of HCC.

Close to 90% of our chronic carriers and HCC patients were Asian, which determined the predominant HBV genotypes detected in this study. Genotype B was found in 48% of the chronic carriers compared with 25% of the HCC patients. In contrast, 67% of our HCC patients had genotype C while only 32% of the chronic carriers had this genotype. The presence of HBV genotype C has been associated with the development of HCC in Asia (9, 10, 31, 32). A study from Shanghai, China, showed that 98% of HCC patients had genotype C (32). In Taiwan, genotype C was associated with older HCC patients, while genotype B was more frequent in young adults and children with HCC (7, 8) However, in Japan, genotype B was more common in older patients with HCC (11, 12). In these latter two reports, HCC patients also had lower rates of HBeAg positivity and lower levels of HBV DNA. Another study from Taiwan showed that genotype C and higher HBV DNA levels were additional risk factors for HCC (14). In addition, HCC patients with either genotype C or genotype B who had BCP mutants and high HBV DNA values were at a higher risk for HCC irrespective of the presence of PC mutants (5, 29, 30). More recently, the presence of T1635 mutation in box α in genotype C patients increased the HCC risk (33). In another study comparing HBV-infected patients with and without HCC, a proline to serine change in codon 38 that encodes the transactivator hepatitis B X protein was associated with the development of HCC (34). In our present report, the HBV genotype was not determined to be a significant risk factor by a logistic regression analysis model adjusted for age, sex, race, genotype, PC mutation and BCP mutation. However, genotype C was found to be a positive confounding factor in the relationship between HCC and BCP mutant (i.e. not adjusting for genotype C, OR for BCP mutants was 16.8, vs. adjusting for genotype C, OR 11.4). In addition, genotype C was found to be a negative confounding factor in the relationship between HCC and PC mutants (i.e. not adjusting for genotype C, OR for PC mutants was 2.52, vs. adjusting for genotype C, OR 3.75).

The presence of HBeAg was reported to predict the development of HCC. In a recent study from Taiwan, the relative risk of HCC was 9.6 in men who were initially positive for HBsAg alone, but was 60.2 among men who were positive for both HBsAg and HBeAg (35). However, the HBeAg tests were performed only at initial recruitment for the study but were not subsequently measured at the time of HCC occurrence. In previous studies, the prevalence rate of HBeAg positivity in patients with hepatitis B-related HCC only was 29% in Taiwan, 13% in South Africa and 15% in the USA (36–38). In these reports, the anti-HBe-positive prevalence rates were 61, 60 and 61% respectively. In our 101 patients with HCC, 70% were anti-HBe positive. In our recent natural history study of 400 HBsAg-positive patients, HBeAg did not appear to have a pathogenetic role in HCC development (26).

The limitations of our study include a single baseline measurement for HBV DNA in both the chronic carriers and in HCC patients. It is well known that fluctuations in serum HBV DNA occur during the course of chronic hepatitis B infection, especially during the exacerbation phases of the disease (39). Also, liver biopsies were not performed in the chronic carriers. However, during a mean 112-month follow-up, these latter patients continued to have normal liver tests and none had liver disease progression. We are continuing to observe these chronic carriers.

In summary, the BCP A1762/T1764 mutation, followed by the PC A1896 mutation had strong correlations with HCC development. Antiviral treatment in these patients before HCC development is warranted in an attempt to delay or prevent the appearance of this lethal malignancy.

## Abbreviations

AFP: α-foetoprotein
Anti-HBe: antibody to HBeAg
BCP: basal core promoter
HBeAg: hepatitis B e antigen
HBsAg: hepatitis B surface antigen
HBV: hepatitis B virus
HBV DNA: hepatitis B viral DNA
HCC: hepatocellular carcinoma
PC: precore
